# Prediction of gastrointestinal bleeding hospitalization risk in hemodialysis using machine learning

**DOI:** 10.1186/s12882-024-03809-2

**Published:** 2024-10-19

**Authors:** John W. Larkin, Suman Lama, Sheetal Chaudhuri, Joanna Willetts, Anke C. Winter, Yue Jiao, Manuela Stauss-Grabo, Len A. Usvyat, Jeffrey L. Hymes, Franklin W. Maddux, David C. Wheeler, Peter Stenvinkel, Jürgen Floege, on behalf of the INSPIRE Core Group, John W. Larkin, Sheetal Chaudhuri, Manuela Stauss-Grabo, Len A. Usvyat, Jeffrey L. Hymes, Franklin W. Maddux, David C. Wheeler, Peter Stenvinkel, Jürgen Floege, Anke Winter, Justin Zimbelman

**Affiliations:** 1 Fresenius Medical Care, Global Medical Office, 920 Winter Street, Waltham, MA 02451 USA; 2 Fresenius Medical Care, Global Medical Office, Bad Homburg, Germany; 3 Fresenius Medical Care AG, Global Medical Office, Bad Homburg, Germany; 4 University College London, London, UK; 5 Dept of Renal Medicine, Karolinska University Hospital, Stockholm, Sweden; 6 Divisions of Nephrology and Cardiology, University Hospital RWTH Aachen, Aachen, Germany

**Keywords:** Bleeding, Gastrointestinal, Hospitalization, Kidney Failure, Predictive Modeling

## Abstract

**Background:**

Gastrointestinal bleeding (GIB) is a clinical challenge in kidney failure. INSPIRE group assessed if machine learning could determine a hemodialysis (HD) patient’s 180-day GIB hospitalization risk.

**Methods:**

An eXtreme Gradient Boosting (XGBoost) and logistic regression model were developed using an HD dataset in United States (2017–2020). Patient data was randomly split (50% training, 30% validation, and 20% testing). HD treatments ≤ 180 days before GIB hospitalization were classified as positive observations; others were negative. Models considered 1,303 exposures/covariates. Performance was measured using unseen testing data.

**Results:**

Incidence of 180-day GIB hospitalization was 1.18% in HD population (*n* = 451,579), and 1.12% in testing dataset (*n* = 38,853). XGBoost showed area under the receiver operating curve (AUROC) = 0.74 (95% confidence interval (CI) 0.72, 0.76) versus logistic regression showed AUROC = 0.68 (95% CI 0.66, 0.71). Sensitivity and specificity were 65.3% (60.9, 69.7) and 68.0% (67.6, 68.5) for XGBoost versus 68.9% (64.7, 73.0) and 57.0% (56.5, 57.5) for logistic regression, respectively. Associations in exposures were consistent for many factors. Both models showed GIB hospitalization risk was associated with older age, disturbances in anemia/iron indices, recent all-cause hospitalizations, and bone mineral metabolism markers. XGBoost showed high importance on outcome prediction for serum 25 hydroxy (25OH) vitamin D levels, while logistic regression showed high importance for parathyroid hormone (PTH) levels.

**Conclusions:**

Machine learning can be considered for early detection of GIB event risk in HD. XGBoost outperforms logistic regression, yet both appear suitable. External and prospective validation of these models is needed. Association between bone mineral metabolism markers and GIB events was unexpected and warrants investigation.

**Trial registration:**

This retrospective analysis of real-world data was not a prospective clinical trial and registration is not applicable.

**Graphical Abstract:**

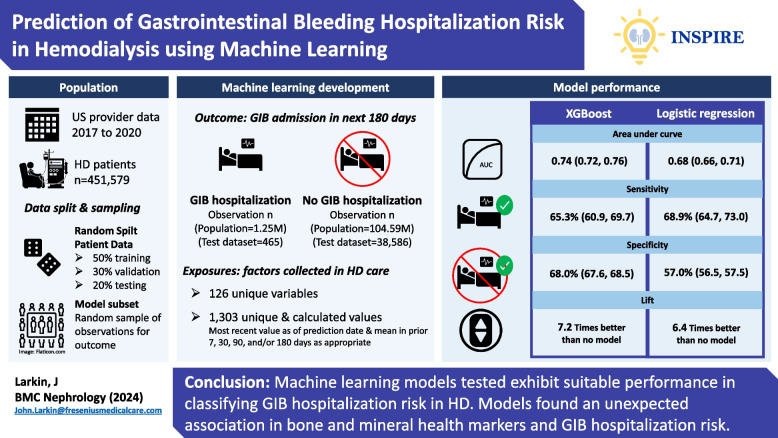

**Supplementary Information:**

The online version contains supplementary material available at 10.1186/s12882-024-03809-2.

## Background

INitiativeS on advancing Patients’ outcomes In REnal disease (INSPIRE) is an academia and industry collaboration set forth to identify critical investigations/models needed to advance the practice of medicine in nephrology. At the inaugural INSPIRE meeting, the Core Group chose major gastrointestinal bleeding (GIB) as a top priority. The consensus was severe bleeding events represent potentially preventable complications occurring more frequently in kidney disease as compared to the general population [1–4].

Major bleeding events have about a 2% to 6% incidence per year in dialysis [5–7], which is more than sevenfold higher than the incidence rate in the general population [8]. Bleeding events differ by modality, with higher rates seen in hemodialysis (HD) versus peritoneal dialysis (PD) [9]. Most bleeding events are due to a gastrointestinal bleed (GIB), with about 20% requiring hospitalization [5, 10]. Incidence of GIB hospitalizations has been increasing over time in the dialysis population [10]. Dialysis patients who experienced a GIB have a 90% higher risk of death occurring any time after the event, a risk that increases with every GIB event [10].

Bleeding risk scores are available for various populations (e.g., GBS [11], HAS-BLED [12], ATRIA [13], HEMORR2HAGES [14], ORBIT [15]), yet have poor performance in dialysis [6, 16, 17]. Machine learning methods were evaluated for classification of major bleeding risk in dialysis, yet have so far shown inadequate performance [6]. The inability to identify the risk for an ensuing bleeding event might be due to the classification for all-cause events, rather than specific types of bleeding events that can have distinct clinical characteristics defining the condition. The INSPIRE Core Group aimed to develop two machine learning models to determine if artificial intelligence-based methods may be able to provide suitable identification of an HD patient’s risk for hospitalization due to a GIB event.

## Methods

### Patient population

We utilized real-world data from adults (age ≥ 18 years) who received ≥ 1 outpatient HD treatment at a national dialysis network (Fresenius Kidney Care, Waltham, United States) during 01-Jan-2017 through 31-Dec-2020.

Project was approved by New England Independent Review Board (Needham Heights, MA, United States; Work Oder# 1–1502098-1) who determined the de-identified data analysis was exempt per United States 45CFR46.104(d)(4). Analysis adhered to the Declaration of Helsinki.

### Outcome and predictor variables

The outcome (dependent variable) was defined as a GIB hospitalization determined from discharge diagnosis ICD10 codes: K22.6, K25.0, K25.2, K25.4, K25.6, K26.0, K26.2, K26.4, K26.6, K27.0, K27.2, K27.4, K27.6, K28.0, K28.2, K28.4, K28.6, K29.0, K62.5, K66.1, K92.0, K92.1, K92.2. At-risk exposure time for outcome prediction was investigated and chosen to be within 180 days after receiving each HD treatment (i.e., prediction date) across the analysis period. The goal was to select a short timeframe that enables actionable interventions while avoiding long-term risk assessments that lack clarity on the benefits of potential actions.

GIB risk factors are uncertain in dialysis. We investigated exposures/covariates (independent variables; Table 1) considering a priori assumptions, as well as common measures captured in care. This permitted exploration based on clinical importance, yet also leveraged the machine learning models’ ability to gain information from large amounts of data [18].

**Table 1:** Exposure variable descriptions considered in modeling

Parameters	Distinct variables	All input variables	Description
**Demographics**			
Age	1	1	Age (years)
Sex	1	1	Male (versus Female)
Race	5	5	Asian, Black, White, other, unknown
Ethnicity	3	3	Hispanic, Not Hispanic, unknown
Height	1	1	Centimeters tall
Dialysis vintage	1	1	Years on chronic dialysis
Marital status	4	4	Single, Married/partner/union, Divorced/separated/widowed, unknown
Dialysis access	2	2	Catheter, Fistula/Graft
**Comorbidities**			
Chronic comorbidity	12	15	Anemias, Hypertension, Diabetes (presence & duration as years since start), Cancer other than skin, Cerebrovascular disease, Chronic obstructive pulmonary disease, Congestive heart failure, Drug or alcohol dependence, Hepatitis, Hyperparathyroidism, Ischemic heart disease, Peripheral vascular/arterial disease (presence)◊
Acute morbidity	4	4	Cardiac arrest, Cardiac dysrhythmias, GI bleed, Infection (presence) ◊
**Environmental**			
Season	4	4	Winter (Dec-Feb), spring (Mar-May), summer (Jun-Aug), autumn (Sep-Nov)
**Laboratories**			
Cell blood counts	9	165	Hemoglobin (weekly), white blood cells, neutrophils, lymphocytes, platelets, monocytes, eosinophils, basophils (monthly), hemoglobin A1C (if has diabetes) (bi-annual)
Chemistry	12	228	Albumin, calcium, corrected calcium, chloride, creatinine, bicarbonate, phosphate, potassium, sodium, blood urea nitrogen, blood urea nitrogen to creatinine ratio, urea reduction ratio (monthly)
Bone factors	2	20	Intact parathyroid hormone (quarterly), total 25OH vitamin D (bi-annual)
Iron indices	2	26	Transferrin saturation, ferritin (quarterly)
**HD Treatment Data**			
Vital signs	12	228	Standing and sitting systolic and diastolic blood pressure, sitting heart rate, temperature (pre-HD & post-HD)
Weights	5	95	Weights last HD (pre-HD & post-HD), estimated dry weight (EDW), removed weight as percent of EDW, removed weight as percent of target to remove
Dialysis delivery	6	114	Treatment time, KECN (effective conductivity clearance of sodium), online clearance Kt/V, Qb, Qd, saline administration
Shortened HD session	14	266	Ended treatment early: against medical advice, patient request, physician request, patient late, complication (clotted access, poor flows, hypotension, technical difficulty, system problem), emergency, hospitalization, unexpected, other, unknown
Rescheduled HD session	5	7	Days since start/end last rescheduled HD, days between rescheduled HD to next session, ≥ 1 rescheduled HD in last 180 days, number of rescheduled HD (last 30, 90, 180 days)
Missed HD treatments	5	7	Days since start/end last missed HD, days between missed HD to next session, ≥ 1 missed HD in last 180 days, number of missed HD (last 30, 90, 180 days)
**Medications**			
In-center medications	10	100	Systemic heparin, heparin catheter lock, IV vitamin D, oral vitamin D (calcitriol, paricalcitol, or ergocalciferol), calcimimetic (etelcalcetide, cinacalcet), erythropoietin stimulating agents, IV iron (dose of medication)
**Events**			
All cause hospitalizations/events	6	6	Days since start/end last hospitalization, ≥ 1 hospitalization in last 180 days, length of stay (days) for last hospitalization, emergency room visit in last 180 days, temporary transfer outside provider in last 180 days

Comorbidities: ◊ ICD10 groupings for all comorbidities available upon request

Laboratories, HD treatment data, in-center medications: Model will consider most recent value for each distinct variable, as well as the minimum, maximum, mean, and difference values (difference in last measure to historic minimum, maximum, and mean values) in the prior 7, 30, 90, and/or 180 days for each distinct variable as deemed appropriate considering data frequency/availability (represented in all variables column of table)

Events: Model will consider most recent value for each distinct variable

**Fig. 1:**
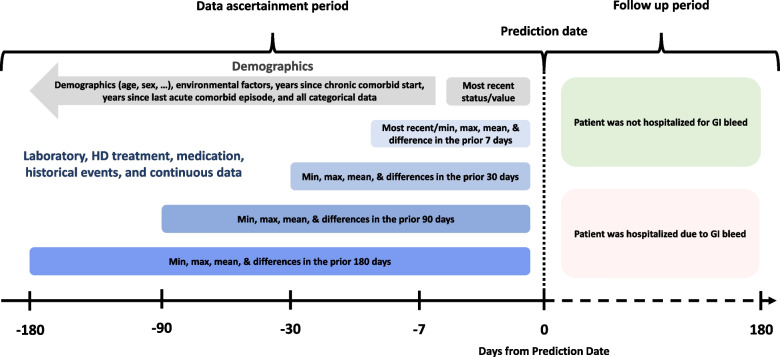
Data ascertainment and outcome follow up timeframes

For each unique exposure (*n* = 126), we included the most recent value/status as of the prediction date; for continuous data, we also included the minimum, maximum, mean, and difference values (difference in last measure to historic minimum, maximum, and mean values) in the prior 7, 30, 90, and/or 180 days (Fig. 1). Erythropoietin doses were converted into epoetin-beta equivalent units using established ratios [19]. Intravenous vitamin D doses were converted to doxercalciferol equivalent units using a 1:1.54 conversion ratio from paricalcitol (i.e. 65% of paricalcitol dose) and a 1:1.375 conversion ratio from calcitriol (i.e. 73% of the calcitriol dose) [20, 21]. Drug doses were considered zero if a patient was not using a medication. Exposure fields with > 60% missingness were excluded due to insufficient data. Imputation of missing data was performed using mean methods for quantitative data and mode methods for qualitative data. Overall, the model assessed 1,303 exposure variables for predictions (unique and calculated variables).

### Data sampling and splitting

**Fig. 2:**
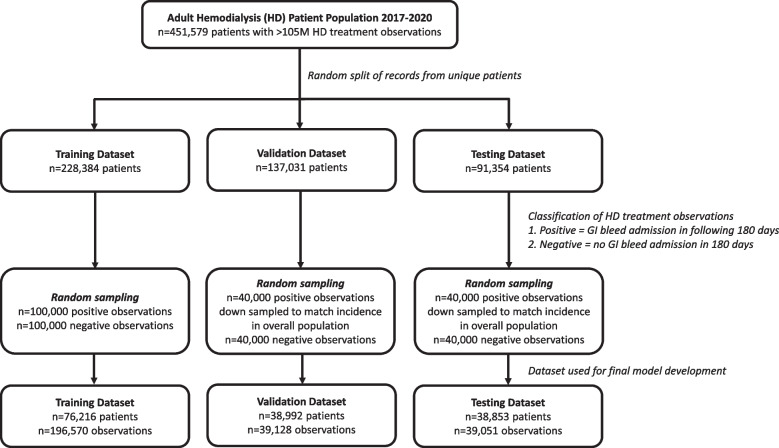
Selection of data for model development

Data was organized for model development by randomly splitting unique patient records into a training (50% of patients), validation (30% of patients), and testing (20% of patients) dataset. In these datasets, each HD treatment observation within 180 days before a GIB hospitalization was classified as a positive observation (i.e., experienced GIB event in next 180 days). All other HD treatment observations were classified as a negative observation (i.e., did not have GIB event in next 180 days). Given the large number of observations (105.84 million HD treatments), we randomly selected a subset of observations for model development, considering samples from the positive and negative observations within these three datasets (Fig. 2). Sampling considered an equivalent number of observations with positive and negative GIB events in the training dataset, and an incidence that matched the overall population in the validation and testing datasets. Repeated observations from unique patients were permitted to be randomly sampled, and could include both positive and negative observations. Sampling considered one randomly selected positive GIB event for each unique patient in the validation and testing datasets to provide equal weight.

### Machine learning models

We used Python version 3.7.7 (Python Software Foundation, Delaware, United States) for machine learning model development in a cloud computing environment (Amazon Web Services, Inc., Seattle, WA). Binary outcome classification models were built using eXtreme Gradient Boosting (XGBoost) and logistic regression methods based on the same datasets and exposures.

XGBoost is a linear and non-linear (decision tree) association model, and logistic regression is a linear association model. [22, 23] Training dataset was used to construct these models that both calculate the likelihood of the outcome as a log-odds value (logarithm of the odds ratio). Outcome classification is based on associations from every possible combination of exposure interactions to maximize information gain, and yields an ensemble of associations (based on log-odds values from decision trees or regression methods). The ensemble models are constructed iteratively (models learn from each iteration, adding new associations to correct errors). Once trained, models used the validation dataset to learn from a different group of patients, and adjust/tune the predictive importance of associations until no further improvements in classification performance were achieved. The final performance was assessed on unseen data in the testing dataset.

### Importance of predictor variables

The importance/meaningfulness of exposures was determined using Shapley (SHAP) values [24, 25] computed using the SHAP python package [26, 27]. SHAP methods determine the effect size (log odds) for each exposure, considering the overall combination of variables, and rank the overall effects on the prediction. SHAP values represent additive explanations of variable importance for linear and non-linear associations in XGBoost model, and the importance for linear associations the logistic regression model.

### Assessment of model performance

Model performance was measured by the area under the receiver operating characteristic curve (AUROC), sensitivity, specificity, accuracy, and balanced accuracy; these were assessed in the training, validation, and testing datasets used for model development. Lift and area under the precision-recall curve (AUPRC) were further assessed in testing dataset. Final model performance was evaluated using unseen testing dataset considering a prediction cutoff threshold of 0.50. The details of the performance metrics are denoted in Additional File 1; Supplementary Methods.

Metrics for AUROC, sensitivity, specificity, accuracy, balanced accuracy and AUPRC compute scores on a scale of 0 (lowest) to 1 (highest). Sensitivity, specificity, accuracy, and balanced accuracy are shown as a percentage. As an example, a model performing at random chance would have an AUROC = 0.5, a balanced accuracy = 50%, and an AUPRC equal to the proportion of positives in the dataset (i.e., incidence of 180-day GIB hospitalization), and a lift value of 1.

## Results

### Patient population characteristics

Incidence of GIB hospitalization within 180 days of a given HD treatment was 1.18% (1,249,108/105,838,571 observations) in the population (patient *n* = 451,579). We split the population into three groups, randomly assigning each distinct patient’s data into a training (*n* = 228,384), validation (*n* = 137,031), or testing (*n* = 91,354) dataset. A random subset of observations was selected for the training (patient *n* = 76,216), validation (patient *n* = 38,992), and testing (patient *n* = 38,853) datasets used to construct the models. This considered down sampling to achieve an equivalent number of observations with positive and negative GIB events in the training dataset, and sampling to match the GIB incidence in the overall population in the validation and testing datasets. Patient characteristics in the subset of data used were reasonably consistent with the overall population, albeit there were some small differences after random splitting/sampling (Table 2). Compared to all patients with a GIB hospitalization, the random subset showed a slightly higher proportion of patients with a black race and arteriovenous HD access. Despite this, the testing dataset exhibited consistent patient characteristics. Compared to all patients without any GIB hospitalization, the random subset showed a higher proportion of patients with a black race, arteriovenous HD access, and diabetes, as well as a longer dialysis vintage. The testing dataset also showed a higher proportion of patients with arteriovenous HD access and diabetes.

**Table 2:** Characteristics of the HD Patient Population, Random Subset of Patients, and Test Dataset of Patients

**Parameter**	**GIB admission mean ± SD OR %**			**No GIB admission mean ± SD OR %**		
	**Population**	**Subset**	**Test Dataset**	**Population**	**Subset**	**Test Dataset**
Patient n	28,644	13,114	465	422,935	146,302	38,586
Observation n	1,249,108	98,269	465	104,589,463	176,480	38,586
Age (years)	67.3 ± 13.0	68.5 ± 12.6	66.9 ± 13.4	62.8 ± 14.6	63.5 ± 14.3	63.8 ± 14.4
Male	55%	55%	57%	58%	58%	58%
White Race	48%	48%	48%	44%	46%	45%
Black Race	28%	31%	27%	22%	27%	22%
Asian Race	3%	3%	3%	2%	2%	2%
Other Race	1%	1%	1%	1%	1%	1%
Unknown Race	20%	17%	21%	30%	23%	30%
Hispanic Ethnicity	9%	10%	9%	10%	11%	10%
Not Hispanic Ethnicity	67%	68%	66%	56%	61%	56%
Unknown Ethnicity	24%	22%	25%	34%	28%	34%
Dialysis vintage (years)	3.9 ± 4.1	4.5 ± 4.1	4.3 ± 4.4	2.3 ± 3.6	3.8 ± 4.0	3.1 ± 3.8
Catheter HD access	28%	19%	28%	43%	25%	33%
Arteriovenous HD access	70%	81%	71%	46%	73%	63%
Diabetes	39%	40%	42%	30%	38%	37%
Ischemic heart disease	24%	24%	25%	15%	19%	19%
GIB (comorbidity)	2%	3.2%	2.8%	0.4%	0.8%	0.7%
Self-reported energy level^a^	45.3 ± 27.8	46.3 ± 27.8	47.7 ± 28.8	46.3 ± 27.3	49.7 ± 27.8	49.4 ± 27.8

^a^KDQOL-36 score for question 10: “Did you have a lot of energy?”

*GIB* Gastrointestinal bleed, *HD* Hemodialysis

### Model performance

**Fig. 3:**
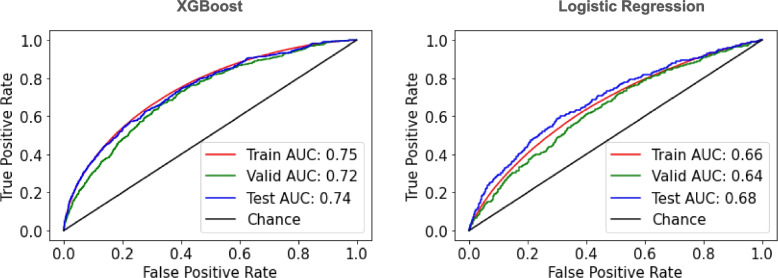
Receiver operating characteristic curve for the XGBoost and logistic regression models

**Fig. 4:**
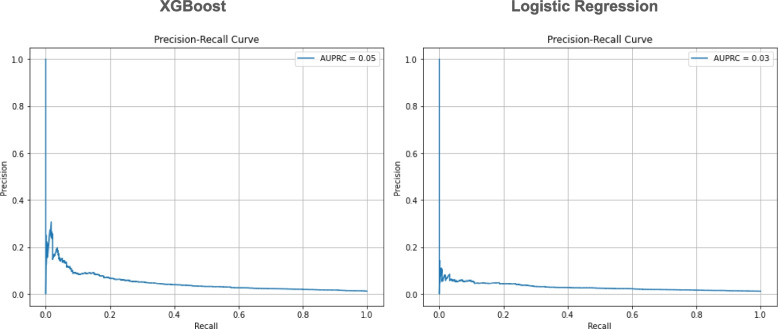
Precision-recall curve for the XGBoost and logistic regression models

**Fig. 5:**
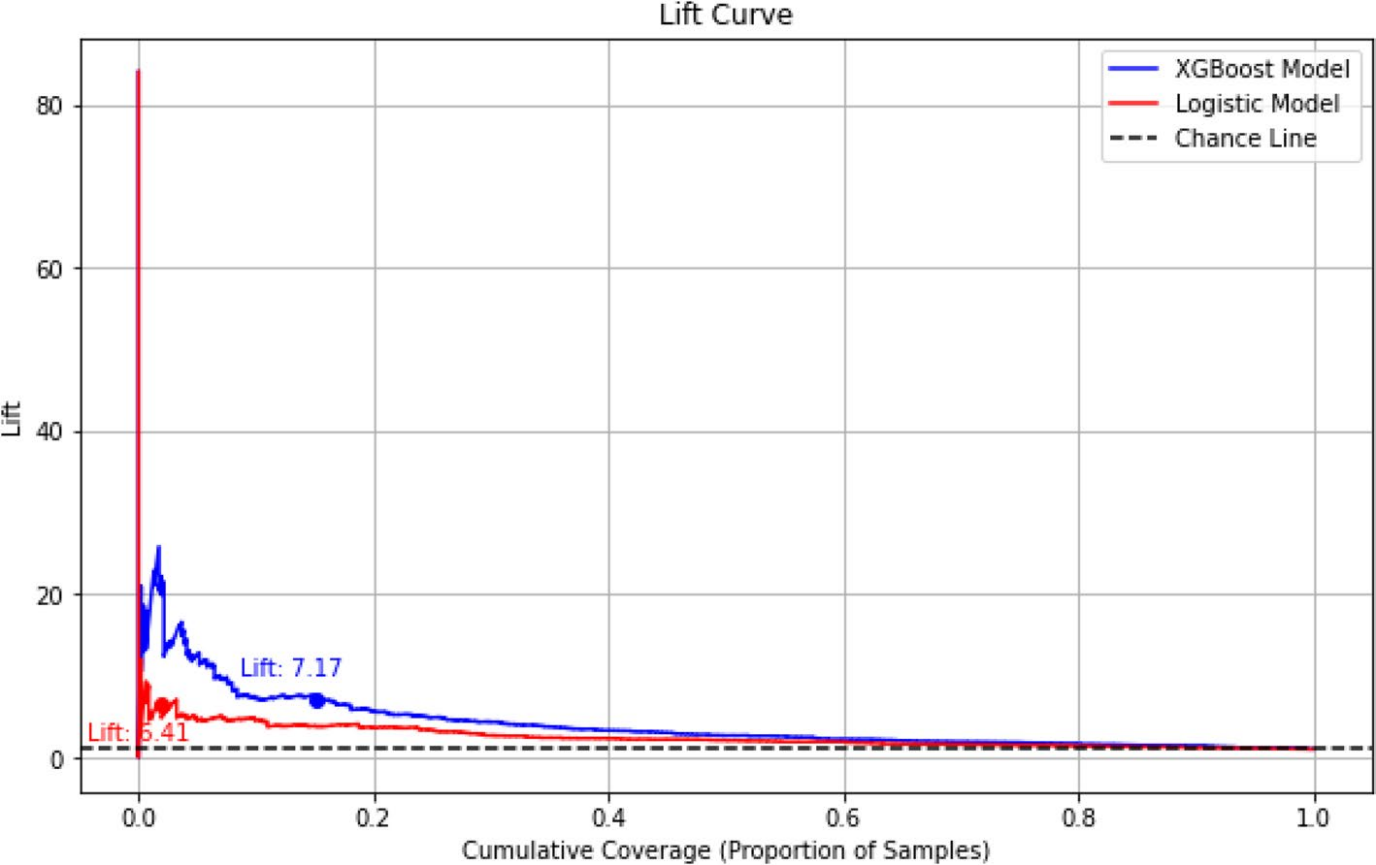
Lift curve for the XGBoost and logistic regression models. Lift values estimate how well the model improves the identification of positive GIB hospitalizations compared to random selection. The x-axis of the lift chart represents the percentage of observations with a positive GIB hospitalization event, while the y-axis represents the cumulative lift above random chance in correctly predicting GIB hospitalization events

Model performance was evaluated on the unseen testing dataset. XGBoost and logistic regression models showed an AUROC of 0.74 (95% confidence interval (CI) 0.72, 0.76) and 0.68 (95% CI 0.66, 0.71) respectively (Table 3, Fig. 3). XGBoost and logistic regression models showed a sensitivity of 65.3% (60.9, 69.7) and 68.9% (64.7, 73.0), a specificity of 68.0% (67.6, 68.5) and 57.0% (56.5, 57.5), and an AUPRC of 0.05 and 0.03 respectively (Fig. 4). Concerning AUPRC, performance at random chance is defined by the incidence of 180-day GIB hospitalization in the population (i.e., 0.012 or 1.2%). In assessment of clinical utility by lift, an estimate of how well a prediction model improves the identification of positive GIB hospitalizations occurring over random selection, XGBoost and logistic regression models showed a lift of 7.2 and 6.4, respectively, suggesting that the models would be 7.2 and 6.4 times more effective in identifying GIB hospitalization as compared to not having any model (Fig. 5).

**Table 3:** Model Performance in Predicting 180-day GI Bleed Hospitalization Risk

Dataset	Training		Validation		Testing	
Patient n	76,216		38,992		38,853	
Observation n	196,570		39,128		39,051	
Incidence of 180-day GIB hospitalization	49.5%		1.12%		1.12%	
Model	**XGBoost**	**Logistic Regression**	**XGBoost**	**Logistic Regression**	**XGBoost**	**Logistic Regression**
AUROC value (95% CI)	0.747 (0.745, 0.750)	0.660 (0.658, 0.663)	0.718 (0.696, 0.741)	0.638 (0.614, 0.663)	0.740 (0.717, 0.763)	0.684 (0.660, 0.709)
Sensitivity % (95% CI)	65.3 (65.0, 65.6)	60.8 (60.6, 61.1)	65.0 (60.9, 69.3)	63.5 (59.2, 67.9)	65.3 (60.9, 69.7)	68.9 (64.7, 73.0)
Specificity % (95% CI)	70.5 (70.2, 70.8)	62.8 (62.5, 63.1)	67.5 (67.1, 68.0)	56.9 (56.4, 57.4)	68.0 (67.6, 68.5)	57.0 (56.5, 57.5)
Accuracy% (95% CI)	68.0 (67.7, 68.2)	61.8 (61.6, 62.0)	67.5 (67.1, 68.0)	57.0 (56.5, 57.5)	68.0 (67.5, 68.4)	57.1 (56.6, 57.6)
Balanced accuracy % (95% CI)	67.9 (0.677, 0.682)	61.8 (61.6, 62.0)	66.2 (64.2, 68.5)	60.2 (58.0, 62.5)	66.7 (64.4, 68.9)	62.9 (60.8, 65.0)

*GIB* Gastrointestinal bleeding, *AUROC* Area under the receiver operating characteristic curve, *CI* Confidence interval

### Predictors of GIB hospitalization

SHAP values estimate the predictive effect size for each variable in the models (Tables 4 and 5). XGBoost showed top three predictors of a GIB hospitalization were a minimum hemoglobin (hgb) in the last 180 days (group mean GIB event = 8.4 g/dL vs no GIB event = 9.1 g/dL), age (group mean GIB event = 66.9 years vs no GIB event = 63.8 years), and total serum 25-hydroxy (25OH) vitamin D levels from the most recent lab (group mean GIB event = 31.6 ng/mL vs no GIB event = 30.3 ng/mL). Logistic regression showed top three predictors of a GIB hospitalization were related to ferritin levels; namely, minimum ferritin in the prior 180 days (group mean GIB event = 744 ng/mL vs no GIB event = 711 ng/mL), ferritin from the most recent lab (group mean GIB event = 958 ng/mL vs no GIB event = 918 ng/mL), and minimum ferritin in the prior 90 days (group mean GIB event = 864 ng/mL vs no GIB event = 834 ng/mL). Top predictors had many consistencies between the two models, yet some distinctions as well. Both models ranked relatively consistent predictive importance for older age, recent all-cause hospitalizations, and disturbances in iron indices, albeit some factors show more predictive importance in one model versus the other. XGBoost showed high importance on outcome prediction for hgb and serum 25OH vitamin D levels, while the logistic regression model showed high importance for heparin and intact parathyroid hormone (PTH).

**Table 4:** Top 25 predictors of 180-Day GI Bleed Hospitalization in the Test Dataset in Reference to XGBoost Model

Top 25 predictors in descending order of importance	XGBOOST mean SHAP value	Logistic regression mean SHAP value	GIB admission mean (SD) OR %	NO GIB admission mean (SD) OR %
Hgb (g/dL): min 180 days	0.42389	0.00034	8.4 (1.4)	9.1 (1.3)
Age (years)	0.32952	0.04080	66.9 (13.4)	63.8 (14.4)
25OH Vitamin D (ng/mL): last lab	0.30093	0.00407	31.6 (13.1)	30.3 (12.8)
Hospitalized in last 180 days (%)	0.16046	0.00005	82%	51%
Dialysis vintage (years)	0.11337	0.00101	4.3 (4.3)	3.2 (3.7)
25OH Vitamin D (ng/mL): difference last lab to mean 180-day	0.06965	0.00002	0.1 (2.1)	0.2 (2.0)
Ferritin (ng/mL): min 180 days	0.06323	0.08896	744 (451)	711 (527)
25OH Vitamin D (ng/mL): difference last lab to max 180-day	0.05865	0.00001	-0.6 (2.6)	-0.5 (2.0)
Dialysis access is fistula/graft	0.05683	0.00000	71%	63%
Hgb (g/dL): last lab	0.05615	0.00030	9.9 (1.6)	10.5 (1.4)
TSAT (%): mean 180 days	0.05190	0.00923	29.4 (10.6)	30.1 (10.4)
25OH Vitamin D (ng/mL): min 180 days	0.04984	0.00394	30.7 (12.6)	29.4 (12.4)
Hgb (g/dL): min 90 days	0.04850	0.00033	8.8 (1.4)	9.4 (1.3)
Ferritin (ng/mL): mean 180 days	0.04553	0.02765	956 (526)	888 (599)
Albumin (g/dL): last lab	0.04403	0.00002	3.5 (0.5)	3.6 (0.5)
TSAT (%): mean 90 days	0.04192	0.01143	28.7 (11.3)	30.4 (11.3)
Ferritin (ng/mL): last lab	0.04160	0.08581	958 (570)	918 (689)
Hgb A1C (%): last lab	0.04028	0.00006	6.4 (1.1)	6.5 (1.0)
Monocytes (%): min 180 days	0.03313	0.00013	5.1 (1.5)	5.3 (1.5)
Cardiac Dysrhythmias	0.03273	0.00001	18%	10%
Days since start of last all-cause hospitalization	0.03160	0.04900	131 (187)	203 (161)
Lymphocytes (%): max 180 days	0.02969	0.00509	21.9 (8.3)	23.0 (9.4)
Post-HD pulse (bpm): max last 90 days	0.02964	0.00512	96.6 (15.4)	93.7 (14.2)
Platelets (1000/mcL): mean 180 days	0.02926	0.01688	202 (72)	206 (73)
Ended HD early against medical advice (%): mean 90 days	0.02866	< 0.00001	0.02 (0.09)	0.02 (0.07)

*GIB* Gastrointestinal bleed, *Hgb* Hemoglobin, *TSAT* Transferrin saturation, *Hgb A1C* Hemoglobin A1C, *HD* Hemodialysis *SD* Standard deviation

**Table 5:** Top 25 predictors of 180-Day GI Bleed Hospitalization in the Test Dataset in Reference to Logistic Regression Model

Top 25 predictors in descending order of importance	XGBoost mean SHAP value	Logistic regression mean SHAP value	GIB admission mean (SD) OR %	NO GIB admission mean (SD) OR %
Ferritin (ng/mL): min 180 days	0.06323	0.08896	744 (451)	711 (527)
Ferritin (ng/mL): last lab	0.04160	0.08581	958 (570)	918 (689)
Ferritin (ng/mL): min 90 days	0.01858	0.05821	864 (523)	834 (608)
Heparin systemic (IU): mean 7 days	< 0.00001	0.05583	126 (731)	162 (950)
Intact PTH (pg/mL): mean 180 days	0.00776	0.05566	453 (418)	458 (394)
Days since start of last all-cause hospitalization	0.03160	0.04900	131 (187)	203 (161)
Heparin systemic (IU): max 7 days	< 0.00001	0.04524	126 (731)	165 (975)
Heparin systemic (IU): min 7 days	< 0.00001	0.04179	126 (731)	159 (933)
Platelets (1000/mcL): min 180 days	< 0.00001	0.04131	163 (65)	174 (70)
Ferritin (ng/mL): max 90 days	0.00085	0.04091	1038 (614)	982 (744)
Age (years)	0.32952	0.04080	66.9 (13.4)	63.8 (14.4)
Intact PTH (pg/mL): last lab	< 0.00001	0.04066	443 (454)	449 (428)
Heparin systemic (IU): mean 30 days	< 0.00001	0.04048	126 (729)	162 (948)
Days since end of last all-cause hospitalization	0.02499	0.03387	126 (187)	198 (161)
Heparin systemic (IU): min 90 days	0.00192	0.03364	84 (639)	130 (826)
Intact PTH (pg/mL): max 180 days	0.02204	0.03338	631 (590)	603 (529)
KECN: max 90 days	0.00125	0.03333	287 (30)	278 (30)
QB (mL/min): difference last HD to min 90-day	< 0.00001	0.03082	75 (65)	69 (65)
Saline (mL): mean 30 days	< 0.00001	0.02899	471 (207)	459 (282)
KECN: max 30 days	< 0.00001	0.02794	279 (31)	271 (30)
Heparin catheter lock (mL): mean 30 days	< 0.00001	0.02765	67 (510)	54 (700)
Ferritin (ng/mL): mean 180 days	0.04553	0.02765	956 (526)	888 (599)
Ferritin (ng/mL): difference last lab to mean 180-day	< 0.00001	0.02725	1.0 (292.8)	29.3 (319.8)
KECN: last HD	0.01001	0.02618	259 (37)	251 (34)
Ferritin (ng/mL): difference last lab to min 180-day	< 0.00001	0.02606	213 (334)	207 (435)

*GIB* Gastrointestinal bleed, *PTH* Parathyroid hormone, *KECN* Effective conductivity clearance of sodium; QB: blood flow

**Fig. 6:**
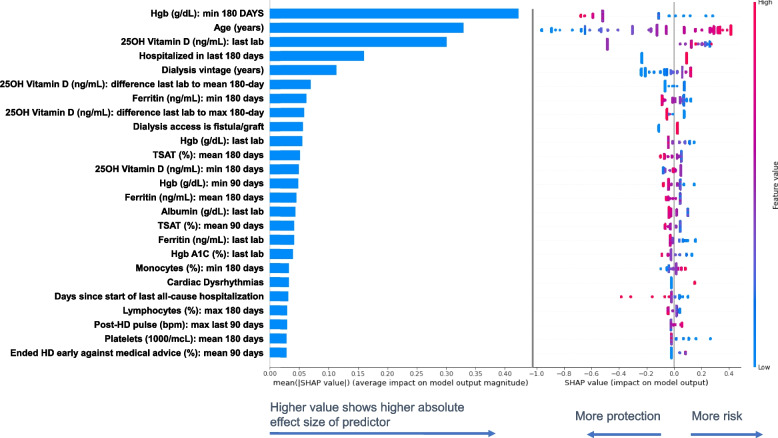
Effect size of the top 25 predictors of 180-day GIB hospitalization in descending order of importance for the XGBoost model. Bar plot on the left panel shows the mean absolute SHAP values that estimate the average effect size of each exposure variable’s contribution to predicting the outcome on the x-axis (calculated from the average absolute value for all predictions). Bee-swarm plots on the right panel show the SHAP value from each prediction as a dot, grouped in a non-overlapping to represent the distribution of the effect size and direction for each exposure variable. Each dot’s position on the x-axis shows variable’s influence on the outcome for that unique prediction (more positive = higher risk or more negative = lower risk/more protection). The color of each dot corresponds to the value for the exposure variable (higher or lower) for that specific prediction. Warmer colors represent higher observed values for that measurement and cooler colors indicate lower values for that measurement. SHAP values are presented in the unit of log odds (i.e. logarithm of the odds ratio). GIB: gastrointestinal bleed; Hgb; hemoglobin; TSAT: transferrin saturation; Hgb A1C: hemoglobin A1C; HD: hemodialysis

**Fig. 7:**
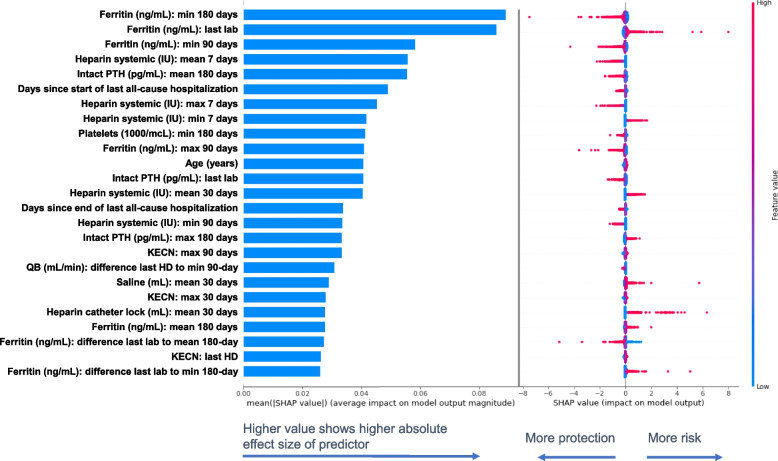
Effect size of the top 25 predictors of 180-day GIB hospitalization in descending order of importance for the logistic regression model. Bar plot on the left panel shows the mean absolute SHAP values that estimate the average effect size of each exposure variable’s contribution to predicting the outcome on the x-axis (calculated from the average absolute value for all predictions). Bee-swarm plots on the right panel show the SHAP value from each prediction as a dot, grouped in a non-overlapping to represent the distribution of the effect size and direction for each exposure variable. Each dot’s position on the x-axis shows variable’s influence on the outcome for that unique prediction (more positive = higher risk or more negative = lower risk/more protection). The color of each dot corresponds to the value for the exposure variable (higher or lower) for that specific prediction. Warmer colors represent higher observed values for that measurement and cooler colors indicate lower values for that measurement. SHAP values are presented in the unit of log odds (i.e. logarithm of the odds ratio). GIB: gastrointestinal bleed; PTH: parathyroid hormone; KECN: effective clearance of sodium; QB: blood flow rate; HD: hemodialysis

Top 25 predictors with the greatest effect on classification of 180-day GIB hospitalization risk are shown in Fig. 6 for XGBoost and Fig. 7 for logistic regression models. Bar charts (left panel) show the mean absolute SHAP value (a non-negative value) representing the magnitude of the effect size for each variable in log odds. Predictors are shown in descending order. The bee-swarm plot on the right panel further illustrates the direction and distribution of effect sizes/SHAP values for each prediction. The x-axis position of each dot indicates the effect size (positive for higher risk, negative for protection), while the color reflects the exposure variable's value (warmer for higher, cooler for lower) for each individual prediction.

To provide an example, the bar charts (mean absolute SHAP value) show minimum ferritin level in the prior 180 days were 7^th^ most important in prediction of 180-day GIB hospitalization risk in the XGBoost model and the 1^st^ most important in the logistic regression model. Bee-swarm plot also shows this ranking, yet further shows dots representing the effect of each individual prediction. The XGBoost bee-swarm plot shows dots with warmer colors for more negative SHAP values (indicating greater protection with higher minimum ferritin levels) and dots with cooler colors for more positive SHAP values (indicating more risk with lower minimum ferritin levels) (Fig. 6). The logistic regression bee-swarm plot also shows warmer colors for more negative SHAP values related to minimum ferritin levels over the prior 180 days (Fig. 7), but cooler colors did not have a remarkable effect size with more positive values (indicating minimal impact on the prediction). Many exposures had a large or small effect size for a specific patient’s prediction, and this can be seen by distributions of SHAP values in bee-swarm plots.

## Discussion

Major GIB events are potentially avoidable, yet underrecognized in HD. To improve methods for early detection, we tested if machine learning could assist in identification of a HD patient’s 180-day GIB hospitalization risk. Two models tested had suitable performance. XGBoost showed higher performance considering AUROC and specificity, yet both models had consistent sensitivity. External and prospective testing appear warranted. Models showed the most important risk factors for GIB hospitalization were older age, disturbances in anemia and iron indices, recent hospitalizations, and bone mineral metabolism markers. Many of the top predictors were anticipated [10, 28], yet the strong associations between serum 25OH vitamin D/PTH and GIB events were unexpected and need further investigation.

We found a 1.2% incidence of 180-day GIB hospitalization, which is consistent with the literature that shows a 2–6% incidence per year. [5–7] Despite a low incidence, experiencing a GIB hospitalization can increase risk of death by 90% in kidney failure [10], emphasizing the need to enhance early detection. GIB can be detected by fecal occult blood tests and endoscopy [29, 30]. However, there is little information to guide screening in the dialysis population and early detection may largely be dependent on a timely referral to a gastroenterologist. Many GIBs can be effectively managed by pharmaceutical regimens or treated during screening procedures, with about 40% of upper GIBs being treated in an outpatient setting [31]. A recent study of > 200,000 hospitalized patients showed kidney failure patients had lower endoscopy rates and higher mortality rates than matched patients without kidney failure [4]. Furthermore, this study showed kidney failure patients with a major GIB who had an endoscopy exhibited lower mortality rates than those who did not receive an endoscopy. This supports the potential benefits of endoscopy for diagnostic evaluation and treatment as it is determined to be appropriate by gastroenterologist evaluation. Notably, kidney failure itself is a significant risk factor for GIB [28, 32, 33]. A study of dialysis patients who received an endoscopy during kidney transplant evaluation showed > 60% of patients had abnormal endoscopic findings [34].

Models are available for assessing risk at the emergency department/hospital. However, these include kidney failure and/or markers altered in kidney disease as inputs, and thus can yield convoluted insights in the dialysis population [35, 36]. GIB risk classification remains a clinical challenge in kidney failure. Glasgow Blatchford score (GBS) has been evaluated for predicting GIB risk and the need for endoscopic intervention in kidney failure patients presenting to the hospital; this model had low to moderate performance (AUROC = 0.63, sensitivity = 81.2%, and specificity = 42.3%) with a GBS cutoff score of ≥ 14 [33]. In comparison, a GBS cutoff score of > 0 (zero) is considered appropriate to define need for endoscopy outside kidney failure [11]. To our knowledge, there are presently no GIB risk models specific to the outpatient kidney failure population. All-cause bleeding risk models have been tested in kidney failure, but are not yet used in care [6, 16, 17, 37]. One all-cause bleeding model (BLEED-HD study) has showed moderate performance (c-statistic = 0.65) in predicting 3-year all-cause bleeding hospitalization risk [37]. Rather than using the outcome of all-cause bleeding, we focused on the most frequent class of bleeding events in a shorter prediction window, which yielded two models that may have suitable performance.

We identified an unexpected and potentially important association between bone mineral metabolism markers and major GIB. More extreme serum 25OH vitamin D values and disturbances appear to be associated with a lower GIB hospitalization risk. A growing body of evidence is emerging on the anticoagulant and antithrombotic actions of serum vitamin D levels and derivative use [38]. Warfarin users have been shown to have a higher GIB risk when serum 25OH vitamin D levels were 30–100 ng/mL versus all other levels [39]. Unadjusted investigations by the INSPIRE Core Group have showed GIB event rates were qualitatively higher in HD when serum 25OH vitamin D levels were 15–50 ng/mL [40, 41]. Models also suggest higher PTH levels may associate with a lower GIB hospitalization risk (PTH measures are top predictors in both models, albeit start with rank at 34^th^ in XGBoost). Dialysis patients with PTH levels < 600 pg/mL have showed significantly increased risk of a major GIB event in a sub-analysis [42].

Concerning pathophysiology of hemostasis, *in-vitro* bench research shows 1,25-dihydroxyvitamin D induces tissue plasminogen activator secretion in rat heart cells [43], down-regulates plasminogen activator inhibitor 1 expression in rat osteoblast cells [44] and human breast cancer cells [45], and down-regulates expression of tissue factors in human leukemia cells [46]. *In-vivo* vitamin D receptor knockout enhances platelet aggregation in mice [47]. Higher serum 25OH vitamin D levels associate with reduced venous thromboembolism risk in the general population [48], and use of 1,25-dihydroxyvitamin D associates with decreased incidence of deep vein thrombosis in prostate cancer [49]. There is a clear need for these interactions to be investigated further, and this is seen in recommendations [50].

The pathophysiology of hemostasis in relation to PTH remains largely undefined, yet involves regulation by the same bone mineral metabolism hormonal axis as 25OH vitamin D, and thus they are inherently intertwined. [50] In-vitro research shows PTH, and its related proteins, can alter plasminogen activator and plasminogen activator inhibitor activities in rat osteoblast and porcine renal epithelial cells. [51, 52] PTH receptors are present on human platelet cells and PTH-related protein can interact with these receptors to enhance platelet activation. [53] Among people with ischemic heart disease on dual antiplatelet therapies, higher serum PTH levels associate with increased platelet aggregation, and a suboptimal response to clopidogrel that did not associate with the effectiveness of other antiplatelet therapies including acetylsalicylic acid or ticagrelor. [54] Furthermore, parathyroidectomy for treatment of uncontrolled secondary hyperparathyroidism associates with decreases in serum platelet activation factor in people on HD. [55] Further research is needed to understand how the bone mineral metabolism axis is associated with and may influence hemostasis and GIB risk in CKD.

Although the models merit further evaluation, there are several limitations to be considered in interpreting findings/predictions. The performance of the models may be suitable and better than existing models, yet further improvements based on prospective evaluations may be worthwhile. GIB is clearly a multidimensional disease, and we did not differentiate bleeding events by lesion location. Bleeding in the upper- and lower-GI system can have differences in etiology, treatment strategies, and outcomes [29, 30, 56]. Predictive models could be designed for specific GIB types, yet this would yield lower incidence rates that may hinder model performance. Associations in exposures show predictive power and may not represent causal relationships. The logistic regression model could be influenced by confounding, which is a limitation with the method when using a more data-driven approach. The XGBoost model is inherently able to manage confounding given the ensemble decision tree method that makes conditional splits, captures complex interactions, and focuses on relevant variables without assuming specific relationships, which may offer an advantage. We chose the default cutoff threshold of 50.0%; this can be adjusted to optimize sensitivity and specificity for a given intervention. Historical data was used for model development and external/prospective evaluations are needed.

## Conclusions

**Fig. 8:**
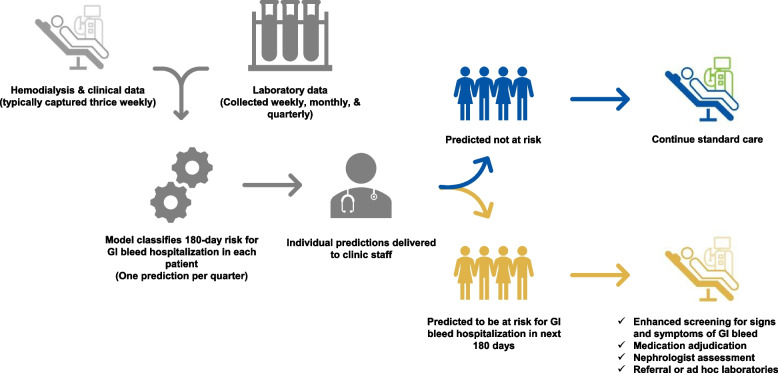
Hypothetical workflow for decision support

Machine learning can be considered for assisting dialysis clinicians in identifying GIB event risk. We found XGBoost outperform logistic regression, yet both models appear suitable. These models offer promising methods for decision support with early detection of an ensuing major GIB, yet they need to be tested in external and prospective evaluations, preferably including randomization of patients or clinics. Figure 8 shows a hypothetical workflow we propose for prospectively testing the developed models in care. We suggest quarterly predictions after comprehensive labs, and reporting including risk classification (e.g., > 80%, ≤ 80% to > 70%, ≤ 70% likelihood of 180-day GIB hospitalization) and a minimum of the top five predictors attributable to each patient’s prediction. As with any developed prediction model chosen to be prospectively tested, performance should be routinely assessed over time and refinements should be performed as needed to maintain performance and improve the model’s utility, especially as it relates to specific interventions warranted. Optimally, reporting could be incorporated into everyday practice and considered at routine visits, just as lab values would be. Risk reporting would be envisioned to assist clinicians in identifying patients who may benefit from a more detailed inquiry on signs/symptoms of GIB, but should not be viewed as a tool for diagnosis of an active GIB warranting rigorous evaluation and additional resource utilization in the absence of appropriate physical clinical evidence to justify a test/procedure.

## Supplementary Information

Additional file 1: Supplementary methods: Detailed description of performance metrics.

## Data Availability

The datasets generated and/or analysed during the current study are not publicly available due to the datasets being captured from a private electronic medical record system that is restricted to use by only authorized employees of Fresenius Medical Care, but are available from the corresponding author on reasonable request. A reasonable request to access the dataset would require an agreement to be established between Fresenius Medical Care and the requestor’s external institution. The model programing can be made available upon reasonable request, which would be considered by the INSPIRE Core Group representing multiple institutions.

## Abbreviations

25OH vitamin D: 25 Hydroxyvitamin D
ATRIA: Anticoagulation and Risk Factors in Atrial Fibrillation score
AUPRC: Area under the precision-recall curve
AUROC: Area under the receiver operating curve
CI: Confidence interval
GBS: Glasgow Blatchford score
GIB: Gastrointestinal bleeding
HAS-BLED: Hypertension, Abnormal renal & liver function, Stroke, Bleeding, Labile INR, Elderly, Drugs or alcohol score
HD: Hemodialysis
HEMORR2HAGES: History of bleeding, Hepatic or renal disease, Alcohol abuse, Malignancy, Older age, Reduced platelet count or function, Hypertension, Anemia, Genetic predisposition, Excessive fall risk, Stroke score
ICD10: International Classification of Diseases-10
INSPIRE: INitiativeS on advancing Patients’ outcomes In REnal disease
ORBIT: Outcomes Registry for Better Informed Treatment of Atrial Fibrillation score
PD: Peritoneal dialysis
PTH: Parathyroid hormone
XGBoost: eXtreme Gradient Boosting model

## References

[1] Sood MM, Larkina M, Thumma JR, Tentori F, Gillespie BW, Fukuhara S, Mendelssohn DC, Chan K, de Sequera P, Komenda P, et al. Major bleeding events and risk stratification of antithrombotic agents in hemodialysis: results from the DOPPS. Kidney Int. 2013;84(3):600–8. 23677245 10.1038/ki.2013.170 PMC3885984

[2] Molnar AO, Bota SE, Garg AX, Harel Z, Lam N, McArthur E, Nesrallah G, Perl J, Sood MM. The risk of major hemorrhage with CKD. J Am Soc Nephrol. 2016;27(9):2825–32. 26823554 10.1681/ASN.2015050535 PMC5004646

[3] Lutz J, Menke J, Sollinger D, Schinzel H, Thurmel K. Haemostasis in chronic kidney disease. Nephrol Dial Transplant. 2014;29(1):29–40. 24132242 10.1093/ndt/gft209

[4] Garg R, Parikh MP, Chadalvada P, Singh A, Sanaka K, Ahuja KR, Aggarwal M, Veluvolu R, Vignesh S, Rustagi T. Lower rates of endoscopy and higher mortality in end-stage renal disease patients with gastrointestinal bleeding: a propensity matched national study. J Gastroenterol Hepatol. 2022;37(3):584–91. 34989024 10.1111/jgh.15771

[5] Holden RM, Harman GJ, Wang M, Holland D, Day AG. Major bleeding in hemodialysis patients. Clin J Am Soc Nephrol. 2008;3(1):105–10. 18003768 10.2215/CJN.01810407 PMC2390984

[6] Nopp S, Spielvogel C, Schmaldienst S, Klauser-Braun R, Lorenz M, Bauer B, Pabinger I, Saemann M, Konigsbrugge O, Ay C. Bleeding risk assessment in end-stage kidney disease: validation of existing risk scores and evaluation of a machine learning-based approach. Thromb Haemost. 2022;122(09):1558–66. 10.1055/a-1754-7551 35098518

[7] Niikura R, Aoki T, Kojima T, Kawahara T, Yamada A, Nakamura H, Inoue K, Morikoshi E, Migita R, Shimizu T, et al. Natural history of upper and lower gastrointestinal bleeding in hemodialysis patients: a dual-center long-term cohort study. J Gastroenterol Hepatol. 2021;36(1):112–7. 32432811 10.1111/jgh.15110

[8] Selak V, Kerr A, Poppe K, Wu B, Harwood M, Grey C, Jackson R, Wells S. Annual risk of major bleeding among persons without cardiovascular disease not receiving antiplatelet therapy. JAMA. 2018;319(24):2507–20. 29946729 10.1001/jama.2018.8194 PMC6583689

[9] van der van EckSluijs A, Abrahams AC, Rookmaaker MB, Verhaar MC, Bos WJW, Blankestijn PJ, Dekker FW, van Diepen M, Ocak G. Bleeding risk of haemodialysis and peritoneal dialysis patients. Nephrol Dial Transplant. 2021;36(1):170–5. 33130878 10.1093/ndt/gfaa216 PMC7771974

[10] Trivedi H, Yang J, Szabo A. Gastrointestinal bleeding in patients on long-term dialysis. J Nephrol. 2015;28(2):235–43. 25185727 10.1007/s40620-014-0132-6 PMC4986509

[11] Blatchford O, Murray WR, Blatchford M. A risk score to predict need for treatment for upper-gastrointestinal haemorrhage. Lancet. 2000;356(9238):1318–21. 11073021 10.1016/S0140-6736(00)02816-6

[12] Pisters R, Lane DA, Nieuwlaat R, de Vos CB, Crijns HJ, Lip GY. A novel user-friendly score (HAS-BLED) to assess 1-year risk of major bleeding in patients with atrial fibrillation: the Euro Heart Survey. Chest. 2010;138(5):1093–100. 20299623 10.1378/chest.10-0134

[13] Singer DE, Chang Y, Borowsky LH, Fang MC, Pomernacki NK, Udaltsova N, Reynolds K, Go AS. A new risk scheme to predict ischemic stroke and other thromboembolism in atrial fibrillation: the ATRIA study stroke risk score. J Am Heart Assoc. 2013;2(3):e000250. 23782923 10.1161/JAHA.113.000250 PMC3698792

[14] Gage BF, Yan Y, Milligan PE, Waterman AD, Culverhouse R, Rich MW, Radford MJ. Clinical classification schemes for predicting hemorrhage: results from the National Registry of Atrial Fibrillation (NRAF). Am Heart J. 2006;151(3):713–9. 16504638 10.1016/j.ahj.2005.04.017

[15] O’Brien EC, Simon DN, Thomas LE, Hylek EM, Gersh BJ, Ansell JE, Kowey PR, Mahaffey KW, Chang P, Fonarow GC, et al. The ORBIT bleeding score: a simple bedside score to assess bleeding risk in atrial fibrillation. Eur Heart J. 2015;36(46):3258–64. 26424865 10.1093/eurheartj/ehv476 PMC4670965

[16] Ocak G, Ramspek C, Rookmaaker MB, Blankestijn PJ, Verhaar MC, Bos WJW, Dekker FW, van Diepen M. Performance of bleeding risk scores in dialysis patients. Nephrol Dial Transplant. 2019;34(7):1223–31. 30608543 10.1093/ndt/gfy387

[17] Satilmis D, Yavuz BG, Guven O, Guven R, Cander B. The effectiveness of Glasgow-Blatchford Score in early risk assessment of hemodialysis patients. Intern Emerg Med. 2022;17(3):753–9. 10.1007/s11739-021-02869-8 34651284

[18] Chaudhuri S, Long A, Zhang H, Monaghan C, Larkin JW, Kotanko P, Kalaskar S, Kooman JP, van der Sande FM, Maddux FW, et al. Artificial intelligence enabled applications in kidney disease. Semin Dial. 2021;34(1):5–16. 32924202 10.1111/sdi.12915 PMC7891588

[19] Stennett A, Mysayphonh C, Kovacevic T, Larkin JW, Guedes M, Moraes TP, Maddux FW, Chatoth D, Pecoits-Filho R, Hymes J: Provider evaluates methoxy polyethylene glycol-epoetin beta in peritoneal dialysis. Nephrol News Issues 2020, 34(9):https://www.healio.com/news/nephrology/20200909/provider-evaluates-methoxy-polyethylene-glycolepoetin-beta-in-peritoneal-dialysis.

[20] Fadem SZ, Al-Saghir F, Zollner G, Swan S. Converting hemodialysis patients from intravenous paricalcitol to intravenous doxercalciferol - a dose equivalency and titration study. Clin Nephrol. 2008;70(4):319–24. 18826857 10.5414/cnp70319

[21] National Kidney F. K/DOQI clinical practice guidelines for bone metabolism and disease in chronic kidney disease. Am J Kidney Dis. 2003;42(4 Suppl 3):S1-201. 14520607

[22] Chen T, Guestrin C. XGBoost: A Scalable Tree Boosting System. In: Proceedings of the 22nd ACM SIGKDD International Conference on Knowledge Discovery and Data Mining. San Francisco: Association for Computing Machinery; 2016. p. 785–94.

[23] Schober P, Vetter TR. Logistic regression in medical research. Anesth Analg. 2021;132(2):365–6. 33449558 10.1213/ANE.0000000000005247 PMC7785709

[24] Shapley LS: “A Value for n-Person Games,” In: H. W. Kuhn and A. W. Tucker, Eds., Contributions to the Theory of Games II. Annals of Mathematics Studies, Princeton University Press, Princeton 1953, 28:307–317.

[25] Štrumbelj E, Kononenko I. Explaining prediction models and individual predictions with feature contributions. J Knowl Inf Syst. 2013;41:647–65.

[26] Lundberg SM, Lee SI. A Unified Approach to Interpreting Model Predictions. In Proc. 31st International Conference on Neural Information Processing Systems 4765–4774. Curran Associates Inc.; 2017.

[27] Lundberg SM, Erion G, Chen H, DeGrave A, Prutkin JM, Nair B, Katz R, Himmelfarb J, Bansal N, Lee S-I. From local explanations to global understanding with explainable AI for trees. Nature Machine Intelligence. 2020;2(1):56–67. 32607472 10.1038/s42256-019-0138-9 PMC7326367

[28] Wasse H, Gillen DL, Ball AM, Kestenbaum BR, Seliger SL, Sherrard D, Stehman-Breen CO. Risk factors for upper gastrointestinal bleeding among end-stage renal disease patients. Kidney Int. 2003;64(4):1455–61. 12969166 10.1046/j.1523-1755.2003.00225.x

[29] Laine L, Barkun AN, Saltzman JR, Martel M, Leontiadis GI. ACG clinical guideline: upper gastrointestinal and ulcer bleeding. Am J Gastroenterol. 2021;116(5):899–917. 33929377 10.14309/ajg.0000000000001245

[30] Strate LL, Gralnek IM. ACG clinical guideline: management of patients with acute lower gastrointestinal bleeding. Am J Gastroenterol. 2016;111(5):755. 27151132 10.1038/ajg.2016.155 PMC12863135

[31] Cooper GS, Kou TD, Wong RC. Outpatient management of nonvariceal upper gastrointestinal hemorrhage: unexpected mortality in Medicare beneficiaries. Gastroenterology. 2009;136(1):108–14. 19010328 10.1053/j.gastro.2008.09.030

[32] Ishigami J, Grams ME, Naik RP, Coresh J, Matsushita K. Chronic Kidney Disease and Risk for Gastrointestinal Bleeding in the Community: The Atherosclerosis Risk in Communities (ARIC) Study. Clin J Am Soc Nephrol. 2016;11(10):1735–43. 27515592 10.2215/CJN.02170216 PMC5053788

[33] Laeeq SM, Tasneem AA, Hanif FM, Luck NH, Mandhwani R, Wadhva R. Upper Gastrointestinal Bleeding in Patients with End Stage Renal Disease: Causes, Characteristics and Factors Associated with Need for Endoscopic Therapeutic Intervention. J Transl Int Med. 2017;5(2):106–11. 28721343 10.1515/jtim-2017-0019 PMC5506410

[34] Sotoudehmanesh R, Ali Asgari A, Ansari R, Nouraie M. Endoscopic findings in end-stage renal disease. Endoscopy. 2003;35(6):502–5. 12783348 10.1055/s-2003-39672

[35] Deshmukh F, Merchant SS. Explainable machine learning model for predicting gi bleed mortality in the intensive care unit. Am J Gastroenterol. 2020;115(10):1657–68. 32341266 10.14309/ajg.0000000000000632

[36] Oakland K, Jairath V, Uberoi R, Guy R, Ayaru L, Mortensen N, Murphy MF, Collins GS. Derivation and validation of a novel risk score for safe discharge after acute lower gastrointestinal bleeding: a modelling study. Lancet Gastroenterol Hepatol. 2017;2(9):635–43. 28651935 10.1016/S2468-1253(17)30150-4

[37] Madken M, Mallick R, Rhodes E, Mahdavi R, Bader Eddeen A, Hundemer GL, Kelly DM, Karaboyas A, Robinson B, Bieber B, et al. Development and Validation of a Predictive Risk Algorithm for Bleeding in Individuals on Long-term Hemodialysis: an international prospective cohort study (BLEED-HD). Can J Kidney Health Dis. 2023;10:20543581231169610. 37377481 10.1177/20543581231169610 PMC10291537

[38] Mohammad S, Mishra A, Ashraf MZ. Emerging Role of Vitamin D and its Associated Molecules in Pathways Related to Pathogenesis of Thrombosis. Biomolecules. 2019;9(11):649. 31653092 10.3390/biom9110649 PMC6920963

[39] Keskin U, Basat S. The effect of vitamin D levels on gastrointestinal bleeding in patients with warfarin therapy. Blood Coagul Fibrinolysis. 2019;30(7):331–6. 31415247 10.1097/MBC.0000000000000841

[40] Larkin JW, Jiao Y, Lama SK, Chaudhuri S, Willetts J, Winter A, Stauss-Grabo M, Usvyat LA, Hymes JL, Maddux FW, Stenvinkel P, Floege J. Higher 25-Hydroxyvitamin D Associates With Gastrointestinal Bleeding Events (Abstract TH-PO837). J Am Soc Nephrol. 2022;33:287.

[41] Larkin J, Jiao Y, Lama S, Chaudhuri S, Willetts J, Winter A, Stauss-Grabo M, Usvayt L, Hymes J, Maddux F, et al. #4768 25 HYDROXYVITAMIN D ASSOCIATES WITH GASTROINTESTINAL BLEEDING IN DIALYSIS. Nephrol Dial Transplant. 2023;38(Supplement_1):i598–9.

[42] Liu J, Guo H, Lin TC, Wetmore JB, Bradbury BD, Gilbertson DT, Nieman K, Peng Y, Sprafka JM, Dluzniewski PJ. Cinacalcet and gastrointestinal bleeding risk in patients receiving hemodialysis. Pharmacoepidemiol Drug Saf. 2022;31(2):141–8. 34363294 10.1002/pds.5337

[43] Puri S, Bansal DD, Uskokovic MR, MacGregor RR. Induction of tissue plasminogen activator secretion from rat heart microvascular cells by fM 1,25(OH)(2)D(3). Am J Physiol Endocrinol Metab. 2000;278(2):E293-301. 10662714 10.1152/ajpendo.2000.278.2.E293

[44] Fukumoto S, Allan EH, Martin TJ. Regulation of plasminogen activator inhibitor-1 (PAI-1) expression by 1,25-dihydroxyvitamin D-3 in normal and malignant rat osteoblasts. Biochim Biophys Acta. 1994;1201(2):223–8. 7947935 10.1016/0304-4165(94)90044-2

[45] Barbosa EM, Nonogaki S, Katayama ML, Folgueira MA, Alves VF, Brentani MM. Vitamin D3 modulation of plasminogen activator inhibitor type-1 in human breast carcinomas under organ culture. Virchows Arch. 2004;444(2):175–82. 14652754 10.1007/s00428-003-0929-5

[46] Koyama T, Shibakura M, Ohsawa M, Kamiyama R, Hirosawa S. Anticoagulant effects of 1alpha,25-dihydroxyvitamin D3 on human myelogenous leukemia cells and monocytes. Blood. 1998;92(1):160–7. 9639512

[47] Aihara K, Azuma H, Akaike M, Ikeda Y, Yamashita M, Sudo T, Hayashi H, Yamada Y, Endoh F, Fujimura M, et al. Disruption of nuclear vitamin D receptor gene causes enhanced thrombogenicity in mice. J Biol Chem. 2004;279(34):35798–802. 15205460 10.1074/jbc.M404865200

[48] Lindqvist PG, Epstein E, Olsson H. Does an active sun exposure habit lower the risk of venous thrombotic events? A D-lightful hypothesis J Thromb Haemost. 2009;7(4):605–10. 19335448 10.1111/j.1538-7836.2009.03312.x

[49] Beer TM, Venner PM, Ryan CW, Petrylak DP, Chatta G, Dean Ruether J, Chi KN, Curd JG, DeLoughery TG. High dose calcitriol may reduce thrombosis in cancer patients. Br J Haematol. 2006;135(3):392–4. 16984385 10.1111/j.1365-2141.2006.06322.x

[50] Disease Kidney. Improving Global Outcomes CKDMBDUWG: KDIGO 2017 Clinical Practice Guideline Update for the Diagnosis, Evaluation, Prevention, and Treatment of Chronic Kidney Disease-Mineral and Bone Disorder (CKD-MBD). Kidney Int Suppl (2011). 2017;7(1):1–59. 30675420 10.1016/j.kisu.2017.04.001 PMC6340919

[51] Takasu H, Guo J, Bringhurst FR. Dual signaling and ligand selectivity of the human PTH/PTHrP receptor. J Bone Miner Res. 1999;14(1):11–20. 9893061 10.1359/jbmr.1999.14.1.11

[52] Catherwood BD, Titus L, Evans CO, Rubin J, Boden SD, Nanes MS. Increased expression of tissue plasminogen activator messenger ribonucleic acid is an immediate response to parathyroid hormone in neonatal rat osteoblasts. Endocrinology. 1994;134(3):1429–36. 8119183 10.1210/endo.134.3.8119183

[53] Ortega A, Perez de Prada MT, Mateos-Caceres PJ, Ramos Mozo P, Gonzalez-Armengol JJ, Gonzalez Del Castillo JM, Martin Sanchez J, Villarroel P, Santiago JL, Bosch RJ, et al. Effect of parathyroid-hormone-related protein on human platelet activation. Clin Sci (Lond). 2007;113(7):319–27. 17501718 10.1042/CS20070010

[54] Verdoia M, Pergolini P, Rolla R, Nardin M, Barbieri L, Schaffer A, Bellomo G, Marino P, Suryapranata H, De Luca G, et al. Parathyroid hormone levels and high-residual platelet reactivity in patients receiving dual antiplatelet therapy with acetylsalicylic acid and Clopidogrel or Ticagrelor. Cardiovasc Ther. 2016;34(4):209–15. 27086085 10.1111/1755-5922.12188

[55] Iatrou C, Antonopoulou S, Andrikopoulos NK, Moutafis S, Tsoufakis G, Movstakas G, Demopoulos CA, Ziroyannis P. The influence of parathyroid hormone on platelet-activating factor (PAF) blood levels in hemodialysis patients. Clin Nephrol. 1995;43(1):60–3. 7697937

[56] Muftah M, Mulki R, Dhere T, Keilin S, Chawla S. Diagnostic and therapeutic considerations for obscure gastrointestinal bleeding in patients with chronic kidney disease. Ann Gastroenterol. 2019;32(2):113–23. 30837783 10.20524/aog.2018.0341 PMC6394262

