# Supplementary material for: Prediction of gastrointestinal bleeding hospitalization risk in hemodialysis using machine learning
Source: BMC Nephrol. 2024 Oct 19;25:366. doi: 10.1186/s12882-024-03809-2 (PMC11490046; doi:10.1186/s12882-024-03809-2)
Supplement: Supplementary file 1 — Additional file 1: Supplementary methods: Detailed description of performance metrics. [file 12882_2024_3809_MOESM1_ESM.docx]

**Supplementary Information:**

**Additional File 1: Supplementary Methods:** Detailed description of performance metrics.

Area under the receiver operating characteristic curve (AUROC), sensitivity, specificity, accuracy, and balanced accuracy were assessed in the training and validation datasets used for model development, and the testing dataset used for determining final model performance. Lift and area under the precision-recall curve (AUPRC) were assessed in the testing dataset for determining final model performance. Final model performance was measured using unseen testing dataset considering a prediction cutoff threshold of 0.50.

AUROC: metric shows the rate of true and false positives classified by model across probability thresholds.

| Definition of true and false positive and negative predictions classified by the model | |
| --- | --- |
| **True positives** | Patients classified as having a GIB event by the model who are in the group with a GIB event |
| **False positives** | Patients classified as having a GIB event by the model who are in the group without a GIB |
| **True negatives** | Patients classified as not having a GIB event by the model who are in the group without a GIB |
| **False negatives** | Patients classified as not having a GIB event by the model who are in the group with a GIB event |

Sensitivity (also known as recall): metric shows the rate of true positives classified by model at a specified threshold and was calculated as follows:

$$\begin{aligned} Sensitivity =\left( \frac{true positives}{(true positives +false negatives)} \right)*100 \end{aligned}$$

Specificity: metric shows the rate of true negatives classified by model at a specified threshold and was calculated as follows:

$$Specificity =\left( \frac{true negatives}{(true negatives +false positives)} \right)*100$$

Accuracy: metric shows the rate of true positives and true negatives classified by model at a specified threshold (i.e., the fraction of correct predictions) and was calculated as follows:

$$Accuracy =\left( \frac{\left( true positives + true negatives \right)}{\left( true positives+true negatives+ false positives+false negatives \right)} \right)*100$$

Balanced accuracy: metric shows the mean of the sensitivity and specificity of model and was calculated as follows:

$$Balanced accuracy =\left( \frac{\left( Sensitivity + Specificity \right)}{2} \right)*100$$

Lift: metric estimates how many times more effective the model is versus standard care by measuring the effectiveness of model compared to random sampling and is calculated as follows:

$$Lift = model precision / proportion of positives in dataset$$

Ultimately, lift values are a measure of how much better the predictions are by the model compared to random guessing. Lift specifically compares the model's ability to correctly identify positive instances (patients in the group with a GIB event) with the expected success rate if no model were used at all (i.e., random selection, proportion of GIB events overall in dataset).

AUPRC: metric measures the ratio of precision for corresponding recall values across probability thresholds.

Metrics for AUROC, sensitivity, specificity, accuracy, balanced accuracy and AUPRC compute scores on a scale of 0 (lowest) to 1 (highest). Sensitivity, specificity, accuracy, and balanced accuracy are shown as a percentage. Lift scores show how many times the model performs over random chance, with a lift value of 2 suggesting the model is twice as effective as random guessing in identifying patients who will experience a GIB event. As an example, a model performing at random chance would have an AUROC = 0.5, a balanced accuracy = 50%, and an AUPRC equal to the proportion of positives in the dataset (i.e., incidence of 180-day GIB hospitalization), and a lift value of 1.
